# Functional diversity in morphologically similar sabre-toothed carnivores

**DOI:** 10.1038/s42003-020-01361-x

**Published:** 2020-10-22

**Authors:** Luke R. Grinham

**Affiliations:** Communications Biology, https://www.nature.com/commsbio

## Abstract

Sabre-toothed carnivores are among the most famed vertebrate fossils in the world. The sabre-tooth ecomorph has been converged upon repeatedly by distantly related species throughout mammalian evolution. Lautenschlager et al. employ a range of biomechanical analyses to investigate the functional diversity of sabre-toothed skulls. Across 66 species, broad functional diversity is recovered with implications for prey specialization and niche partitioning, despite being morphologically convergent.

Few fossil species can compete with the allure and grandeur of dinosaurs, but a strong contender is the sabre-toothed cat. *Smilodon fatalis* is immortalised today in pop culture, childrens’ toys and the hearts of fossil lovers everywhere. However, it was not the only sabre-toothed vertebrate to have ever lived. As is often the case with body forms in biology, the sabre-toothed skull has evolved multiple times in independent species, a process known as evolutionary convergence. Convergent evolution results in animals achieving similar shapes or functions, often with convergent animals occupying similar niches. In the case of the sabre-toothed skull, this can be simplified to oversized upper canine teeth and enhanced jaw gape, and this ecomorph is currently absent in modern fauna. The functional convergence of sabre-toothed carnivores has not been assessed in mechanical detail, until now.

Lautenschalger et al.^1^ conducted a detailed biomechanical analysis of the skulls of 66 sabre-, scimitar- and dirk-toothed fossil vertebrates, spanning 265 million years of evolutionary time from the Permian to the Pleistocene. Using finite-element models, biomechanical simulations, geometric morphometrics and phylogenetic comparative methods, the function and evolution of the sabre-toothed ecomorph was investigated.

Gape angle, mandibular bending strength and bite efficiency were found to vary considerably across taxa and convey different information about hunting style and skull function. For example, within gorgonopsians, the authors suggest a specialisation to smaller prey is indicated by an effective gape angle below 60°. Across all species, bending strength of the mandible does not correlate with jaw gape, but interestingly the rate of jaw bending evolution varies a lot across groups. A significant correlation between canine length and jaw length indicates that maximum canine penetration is more important in the hunting styles of sabre-toothed animals than prey size.

These results, along with others reported in the paper, suggest that there was a wide variety of functional convergence in sabre-toothed vertebrate ecomorphology not apparent with surface level observations of these skulls. The morphological convergence in these species does not correlate with the breadth of functional diversity reported by the authors in this exciting new study.
